# Central venous-to-arterial carbon dioxide difference in the early postoperative care following liver transplantation

**DOI:** 10.1186/2197-425X-3-S1-A821

**Published:** 2015-10-01

**Authors:** I Saez, J Sáez, M Talayero, N González, M Catalán, JÁ Sánchez Izquierdo, JC Montejo

**Affiliations:** Hospital 12 de Octubre, Madrid, Spain; Hospital Infanta Leonor, Madrid, Spain

## Introduction

The early postoperative period after liver transplantation is characterized by a complex hemodynamic situation, due to the interaction of multiple factors, as the persistence of the hyperdynamic circulation or the increase of lactate blood levels because of the impaired hepatic clearance.The central venous-to-arterial carbon dioxide difference (P_CVA_CO_2_ gap) has been proposed to better identify patients with persistent global hypoperfusion.

## Objectives

To analyze the ability of the P_CVA_CO_2_ gap in the hemodynamic management after liver transplantation.

## Methods

Prospective observational study including all patients admitted to the ICU after liver transplantation over a 4-months study period. Demographic variables (sex, age, cause of transplantation and severity scores), hemodynamic variables, and those related to graft function and postoperative complications were recorded.Data statistical analysis was done using SPSSâ software. Quantitative variables are expressed as mean with standard deviation (SD) and qualitative variables as absolute and relative frequencies. The statistical significance of the proportion comparison was addressed by the chi-square test or Student´s t-Test with a statistical significance p < 0.05.

## Results

During the study period, 24 patients were admitted to the ICU after a liver transplantation (80% male) with a mean age of 55,9 ± 10 years. Mean MELD score was 15 ± 9; Mean Child-Pugh was 8 ± 3. Mean ICU stay was 8 ± 8 days, with an overall mortality of 8%. Mean hospital stay was 18 ± 8 days. Early graft dysfunction incidence was 12,5%. Delayed extubation (>24H) incidence was 16% meanwhile acute renal incidence was 58% (14% AKIN I, 21% AKIN II y 64% AKIN III). Two patients (8%) presented infectious complications and four 4(16%) developed seizures. During the 72 first hours of ICU stay, 13 patients (54% of the sample) presented a P_CVA_CO_2_ gap > 6 mmHg, simultaneously with a cardiac index >3 l/min/m^2^ and a mixed venous oxygen saturation >60%. Delayed extubation was associated with an elevated P_CVA_CO_2_ gap in 30,8% of the cases vs. 0% in those with a normal P_CVA_CO_2_ gap (p = 0.98). An elevated P_CVA_CO_2_ gap was associated with longer ICU stay (11,2 vs 4,2, p = 0.038) and hospital stay (19,6 vs. 15,6, p = 0.334).

## Conclusions

In the early postoperative care after liver transplantation, the P_CVA_CO_2_ gap is a feasible hemodynamic parameter that could predict a worse clinical outcome.
